# Uncovering common pathogenic transcriptional dysregulations in Silver-Russell syndrome

**DOI:** 10.1186/2194-7791-1-S1-A13

**Published:** 2014-09-11

**Authors:** Florian Bohne, Ursula Martiné, Matthias Begemann, Thomas Eggermann, Thorsten Enklaar, Dirk Prawitt

**Affiliations:** Center for Pediatrics and Adolescent Medicine, University Medical Center, Mainz, Germany; Institute of Human Genetics University Hospital, Aachen, Germany

Silver-Russell syndrome (SRS) is a rare developmental disorder, presenting with marked intrauterine and postnatal growth retardation. The heterogeneous associated molecular defects in approximately 40% of SRS patients are due to epigenetic changes in the imprinting control region 1 (ICR1) that regulates the monoallelic expression of *IGF2* and *H19* in *cis*. Nevertheless a significant number of SRS patients have epigenetic alterations in other imprinted regions instead, like in cases with maternal uniparental disomy for chromosome 7 (upd(7)mat). Using primary fibroblasts from SRS patients with upd(7)mat and a unique case with upd(11)mat, we can show dysregulation of imprinted genes not located on the chromosomes 7 or 11 respectively, but on other chromosomes, suggesting a functional interaction between imprinted genes throughout the genome. 4C analyses for the ICR1 have shown that this interaction is unlikely to be based on chromatin interactions, but rather due to effects of the gene products themselves. The most likely transfactors mediating the transcriptional regulation of other imprinted genes are long non-coding RNAs. To determine which of these function as transfactors for the transcriptional regulation of other imprinted genes, we performed *knock-down* assays and monitored the transcriptional consequences on other imprinted genes. Our preliminary analyses also show, that common dysregulation patterns for imprinted genes throughout the genome can be described in SRS patients with different epigenetic alterations, suggesting that these patterns are associated with the clinical phenotype.

Funded by BMBF 01GM1114D.

